# Pharmacological Effect of* Caulophyllum robustum* on Collagen-Induced Arthritis and Regulation of Nitric Oxide, NF-*κ*B, and Proinflammatory Cytokines In Vivo and In Vitro

**DOI:** 10.1155/2017/8134321

**Published:** 2017-12-31

**Authors:** Qiu-hong Wang, Shao-wa Lv, Yu-yan Guo, Ji-xin Duan, Shu-yu Dong, Qiu-shi Wang, Feng-ming Yu, Hong Su, Hai-xue Kuang

**Affiliations:** ^1^Heilongjiang University of Chinese Medicine, Harbin 150040, China; ^2^Guangdong Pharmaceutical University, Guangzhou 510006, China

## Abstract

*Caulophyllum robustum* Maxim* (C. robustum)* has commonly been used as traditional Chinese medicine for the treatment of rheumatic pain and rheumatoid arthritis (RA) in China. This paper first investigated the anti-inflammation effect of* C. robustum* extraction (CRME) on RAW264.7 cells stimulated by lipopolysaccharide (LPS) and gene expression levels of inflammatory factors. Moreover, we first evaluated the anti-RA effects of CRME using collagen-induced arthritis (CIA) in DBA/1J mice, and the incidence, clinical score, and joint histopathology were evaluated. The levels of IL-1, IL-6, TNF-*α*, and PGE2 inflammatory factors in sera of mice were detected by enzyme-linked immunosorbent assay. The expression of NF-*κ*B p65 in the joint was tested by immune histochemical technique. The results showed that, compared with the model group, CRME significantly improved symptoms of the arthritis index, limb swelling, and histological findings by decreasing synovial membrane damage, the extent of inflammatory cell infiltration, and the expansion of capillaries in CIA mice. The results also showed that CRME can reduce the levels of IL-1, IL-6, TNF-*α*, and PGE2 and inhibit the expression of NF-*κ*B p65. All these results indicated the anti-inflammatory efficacy of CRME as a novel botanical extraction for the treatment of RA.

## 1. Introduction

As an autoimmune disease, human rheumatoid arthritis (RA) is a major cause of disability characterized by chronic inflammation and joint damage. The pathogenesis of RA is a complex process involving synovial cell proliferation and fibrosis, pannus formation, and cartilage and bone erosion [1, 2]. To date, it has been found that the joint destruction is caused by several mechanisms including the overexpression of inflammatory response cytokines and transcription factors relevant to RA inflammation during the occurrence of RA [3]. In RA, the transcription factor nuclear factor-kappa B (NF-*κ*B) is overexpressed in the inflamed synovium [4, 5]. It participates in the transactivation of various genes and inducible nitric oxide (iNOS) [6], which mediate immune and inflammatory responses [7]. It can activate and be activated by proinflammatory cytokines, such as Tumor Necrosis Factor-alpha (TNF-*α*), Interleukin- (IL-) 1, Prostaglandin E (PGE), and IL-6, resulting in a positive regulatory circle that causes amplification of local inflammatory responses [8]. TNF-*α* stimulates synovial fibroblasts to express intercellular adhesion molecules, which increases the migration of leukocytes into the RA joints. It is a central factor in the pathogenesis of RA as it amplifies inflammation and causes joint damage. IL-6 is also known as another proinflammatory cytokine that closely contributes to vascular endothelial growth and that stimulates the growth of blood vessels, including those in hypertrophy of joints. IL-1 is another key mediator involved in bone resorption and cartilage destruction that can modulate the production of nitric oxide and PGE2. PGE2 can act to mediate hyperalgesia-induced sensitizing pain receptors and induce fever [9]. Relieving inflammation and reducing proinflammatory cytokine overexpression are therefore regarded as one of the major approaches to help prevent the progressive damage to RA joints.

Historically,* C. robustum* is a traditional Chinese herb used in the treatment of RA [10]. It is acrid and bitter in taste and warm in nature. The Dictionary of Chinese Medicinal Plants records that* C. robustum* roots and rhizomes have the ability to expel wind, promote blood circulation, and regulate the flow of vital energy to stop pain. It is mainly used for external injuries, rheumatism, and RA treatment. Pharmacological study showed that its decoction inhibits tissue edema caused by croton oil, suppresses capillary permeability induced by acetic acid, and suppresses cotton pellet granuloma [11]. Modern studies have demonstrated that alkaloids and triterpenoid saponins seem to contribute most of the physiological anti-inflammatory activity [12]. Among them, Caulosides A~D could dramatically suppress the expression of proinflammatory factors, for instance, TNF-*α*, IL-1*β*, and IL-6 [13–15]. Magnoflorine has shown activity against FCA-induced pyrexia [16]. However, some potential toxic and side effects of* C. robustum* have been found. For its convenient clinical use, we improved the extraction technique and obtained CRME with lower toxicity (LD50 is 4.6 g).

We set out to investigate the anti-inflammation effect of CRME on RAW264.7 cells stimulated by LPS. Then, collagen-induced arthritis (CIA) by the immunization of DAB/1 mice with type II collagen in complete Freund's adjuvant was used for activity evaluation in vivo. The levels of IL-1*β*, IL-6, TNF-*α*, and PGE2 in sera of the CIA rats and of NF-*κ*B protein were determined in synovial tissue. All the above were helpful to our investigation into the therapeutic effects of CRME on RA and its possible mechanism.

## 2. Materials and Methods

### 2.1. Sample Extraction and Isolation of* C. robustum*

**Figure 1:**
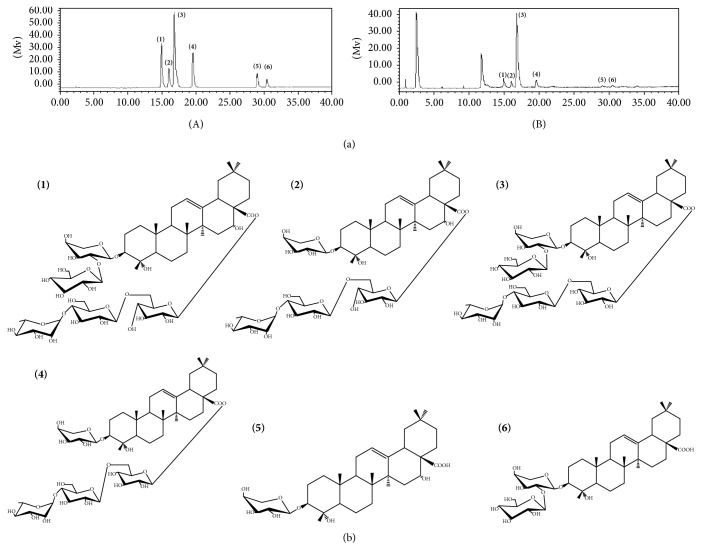
(a) HPLC–ELSD chromatogram of six marker compounds in CRME. HPLC–ELSD chromatogram of mixture of standard compounds (A). HPLC–ELSD chromatogram of six marker compounds in CRME (B). (b) Structure of six marker compounds. Cauloside H** (1)**. Leonticin D** (2)**. Cauloside G** (3)**. Cauloside D** (4)**. Cauloside B** (5)**. Cauloside C** (6)**.

*C. robustum* was purchased from Heilongjiang province of China and authenticated by Professor Shaowa Lv (College of Pharmacy, Heilongjiang University of Chinese Medicine). A specimen (20150301) of it was deposited in the College of Pharmacy, Heilongjiang University of Chinese Medicine. Its roots and rhizomes were extracted twice with 70% ethanol (v/v). The filter was combined and reclaimed and then loaded onto an AB-8 macropore resin column and eluted with pure water and 70% ethanol (v/v). The 70% ethanol eluate was concentrated to dryness (the powder was named CRME). We determined its main chemical components by high-performance liquid chromatography (HPLC). The result showed that there were six components in CREM, including Cauloside H, Leonticin D, Cauloside G, Cauloside D, Cauloside B, and Cauloside C and their content was 6.21%, 5.14%, 28.88%, 10.19%, 3.68%, and 2.26%, respectively (Figures 1(a) and 1(b)).

### 2.2. Drugs and Reagents

The RAW264.7 cell line was purchased from the Cell Resource Center, Peking Union Medical College. gDNA and cDNA Remover were purchased from TOYOBO Co., Osaka, Japan. Trizol reagent was purchased from Tiangen, Beijing, China.

LPS, vine type II collagen, Freund's incomplete adjuvant (IFA), and Freund's complete adjuvant (CFA) were purchased from Sigma (USA, St. Louis, MO). IL-1*β*, IL-6, TNF-*α*, PGE2, and NO were purchased from R&D system in the USA. NF-*κ*Bp65 was purchased from Wuhan Boster Biological Engineering Co., Ltd. The rabbit-anti-mouse second antibody and DAB were purchased from Beijing Chinese Fir Golden Bridge Biotechnology Co., Ltd.

Triptolide- (Tri-) tablets were purchased from Broad medicine Yellowstone Feiyun Pharmaceutical Co., Ltd., lot number: 20130601, standard: 10 mg. Methotrexate (MTX) tablets were purchased from Shanghai Sym. Pharma Co., Ltd., lot number: 03614040, standard: 2.5 mg. To determine the therapeutic efficiency of CRME for the treatment of RA, we chose MTX and Tri as positive medicines. MTX is the first-line therapy for this disease, and Tri is isolated from Tripterygium Wilfordii Hook f., which is a kind of Chinese medicinal plant used to ameliorate the symptoms of rheumatic diseases [17].

### 2.3. Cell Cultures and Their Use to Test CRME

RAW264.7 cells were maintained in DMEM containing 10% fetal bovine serum (FBS) and 1% penicillin streptomycin (PS) at 37°C in a 5% CO2 incubator. Before starting any experiment, RAW264.7 cells were seeded in culture plates and maintained for 24 h until approximately 70%–80% confluence. RAW264.7 cells were stimulated with LPS of* Escherichia coli* (serotype, 055: B5) at a concentration of 1 *μ*g/ml for establishing the inflammatory cell model. RAW264.7 cells were incubated with CRME by the addition of different concentrations of the former (0.250 mg/mL, 0.125 mg/mL, and 0.063 mg/mL) for 1 h and then treated with 1 *μ*g/mL LPS for an additional 24 h. After 24 h, the cell culture supernatant was collected to detect NO (nitric oxide) by Griess reagent assay. Total RNA was isolated immediately after harvesting the RAW264.7 cells for RT-PCR as described below. Unstimulated RAW264.7 cells were treated as a negative control.

### 2.4. RNA Extraction and RT-PCR Analysis

Total RNA was separated from RAW264.7 cells by Trizol reagent according to the protocol. For cDNA synthesis, total RNA (2 *μ*g/sample) was reverse transcribed with ReverTra Ace qPCR RT Master Mix with the gDNA Remove. The cDNA was then amplified with THUNDERBIRD SYBR qPCR Mix. The primer sequences were as follows: for IL-1*β*, forward primer (FP) = (5′-CAGGATGAGGACATGAGC ACC-3′) and reverse primer (RP) = (5′-CTCTGCACACTCAAACTCCAC-3′), for TNF-*α*, FP = (5′-GG GAG CAAAGGTTCAGTGAT-3′) and RP = (5′-CCTGGCCTCTCTACC TTGTT-3′), for IL-6, FP = (5′-CTGACAATA TGAATG TTGGG-3′5′-TCCAAGA AACATCTG GC TAGG-3′); for GAPDH, FP = (5′-GTCATTGAGAGCAATGCCAG -3′) and RP = (5′- GTGTTCCT ACCCCCAATGTG -3′). The PCR amplification conditions were as follows: initial denaturation at 95°C for 1 min; 40 cycles of denaturation and extension at 95°C for 15 s. Amplified products were separated by 1% agarose gel electrophoresis and visualized with ethidium bromide staining. The results are representative of three independent experiments.

### 2.5. Animals, Induction of CIA, and Compound Admini-Stration

DBA/1 mice (half male and female, 6~8 weeks old) were purchased from HFK Bioscience Co., Ltd. (Beijing, China, Certificate: 2010A050). All mice were maintained in a room with a temperature of 25 ± 2°C and humidity of 60%. Mice drank water freely and were given 12 h of light each day. Bedding was kept dry. All animal care and treatment procedures were approved by the committee of Experimental Animal Administration of Heilongjiang University of Chinese Medicine.

Forty-two DBA/1 mice were divided into seven groups with identical numbers of mice (*n* = 6). One of the groups served as “normal,” whereas the other 6 groups were subjected to CIA induction. Bovine type II collagen was dissolved in 0.1 M acetic acid, mixed to 2 mg/ml mixed liquor, and incubated overnight at 4°C. The mixture and an equal volume of CFA were emulsified at low temperature. The DBA/1 mice were subcutaneously immunized at 1-2 cm from the base of the tail with 100 *μ*L hybrid emulsion (containing 100 *μ*g of bovine type II collagen), and this was treated as the first immunization on day 0. Mice were boosted intraperitoneally with 100 *μ*g bovine type II collagen emulsified in an equal volume of IFA on day 21, and this was treated as the second immunization.

CRME, Tri tablets, and MTX tablets were separately dissolved in distilled water and administered orally once per day for 30 days, from day 22 to day 52. The groups are as follows: normal group (Normal), CIA model group (Vehicle), Tri group 11.3 mg/kg, MTX group 0.9 mg/kg, CRME group (L group) 24.8 mg/(kg·day), CRME group (M group) 49.7 mg/(kg·day), and CRME group (H group) 99.4 mg/(kg·day). The dosage selection for the present study was according to the LD50 (4.6 g/kg) for CRME from our previous study and reference to classical Chinese medicine books and literature. For example, Guizhou folk medicine records that 5 g* C. robustum* powder taken with wine has the most significant effect. In addition, experiments show that* C. robustum* ethanol extract (69.23, 34.61, and 17.31 mg/kg) inhibits the swelling of the joint and decreases the spleen index and the level of inflammatory factors [18].

### 2.6. Morphologic and Immune Studies

Mice were observed once every 3 days after primary immunization. Arthritis severity was evaluated by arthritis index, arthritis incidence, and percentage of arthritic limbs as assessed by two independent, blinded observers. All four limbs of the mice were evaluated and scored from 0 to 4 as follows: 0, normal; 1, 1~2 toe joints swelling; 2, the whole ball with mild swelling and redness; 3, severe swelling and redness of the whole ball; 4, overall deformation and/or joint stiffness. The arthritis index of all four limbs was the total score, and the highest score for each mouse was 16 points. When the score of a paw was more than 2, the mice were considered to suffer from RA. The volume of arthritic limbs was detected with a digital plethysmometer (LE 7500, Panlab, Spain). According to the digit volume of mice and the following formula, the limb swelling of mice was calculated:

(1) Sweling degree%=Arthritic limb volum−Normal limb volumeRight rear normal volume×100%.

There is a close connection between the aggressive progression of RA and the loss of body mass. After the secondary immunization, we detected the changes in body weight once every 3 days in order to evaluate CRME for the treatment of RA. The weight of individual CIA mice was calculated with the following formula:

(2) Change of body weight%=body weightday 31 of arthritisbody weightday 1 of arthritis×100%.

Mice were sacrificed by cervical dislocation after a month of oral administration. The spleen and thymus were obtained, fur and blood on the surface of the viscera were rinsed clean with normal saline, and they were weighed with an analytical balance. We measured the wet weight of the immune organs to preliminarily assess CRME for treatment of RA. The computation formula is as follows:

(3) Viscera index%=Weight of spleenor thymusWeight of mice×100%.

### 2.7. Enzyme-Linked Immunosorbent (ELISA) Assay

On the 52nd day, 1 mL of blood was collected from each mouse's eye socket vein after more than 12 h fasting. Then, the blood samples stood at room temperature for 20 min. Serum was separated from the rest of the blood using a high-speed centrifuge (H2050R, China) at 10000 rpm for 10 min, and IL-1*β*, IL-6, TNF-*α*, and PGE2 levels were measured with ELISA kits. According to the manufacturer's instructions, the processed samples were measured for OD value at 450 nm. There is a certain linear relationship between cytokine concentrations in mice and the OD value, which can be determined by acknowledgement of standard curve sample concentrations of cytokines in mice, and, then, the data were analyzed to sum up the influence of CRME on the inflammatory factors.

### 2.8. Histological

On the 52nd day, the mice were sacrificed. The hind limbs and immune organs, such as spleen and thymus, were fixed immediately in 4% buffered formalin solution, decalcified, and embedded in paraffin, after which they were divided into 6 *μ*m pieces. The left hind limbs and visceral organs were treated with Hematoxylin-Eosin (HE) staining for morphological examination, while the right hind limbs were used for immunohistochemistry with NF-*κ*Bp65. A Motic 3000 micrograph system served as radiography at 400 times, and Image-pro plus 6.0 pathological Image was used to analyze positive expression quantitatively. The expression of protein was represented by integral optical density (IOD), namely, integral optical density (IOD) = positive area × average optical density. Each case was analyzed randomly in two vision fields at high magnification, and, then, the relative expression was averaged, for which there were 3 cases in each group.

### 2.9. Statistics

The software SPSS version 19.0 for Windows was applied for statistical analysis. Measurement data were expressed as the mean ± SD, and the two-way ANOVA test followed by least-significant differences (LSD) test was used to compare between groups; count data were expressed as percentages and compared with the chi-square test between groups. Statistical significance was set at ^*∗*^*P* < 0.05, and extreme significance was set at ^#^*P* < 0.01.

## 3. Results and Discussion

### 3.1. Levels of Nitric Oxide (NO) in Culture Supernatant of RAW 264.7 Cells

**Figure 2:**
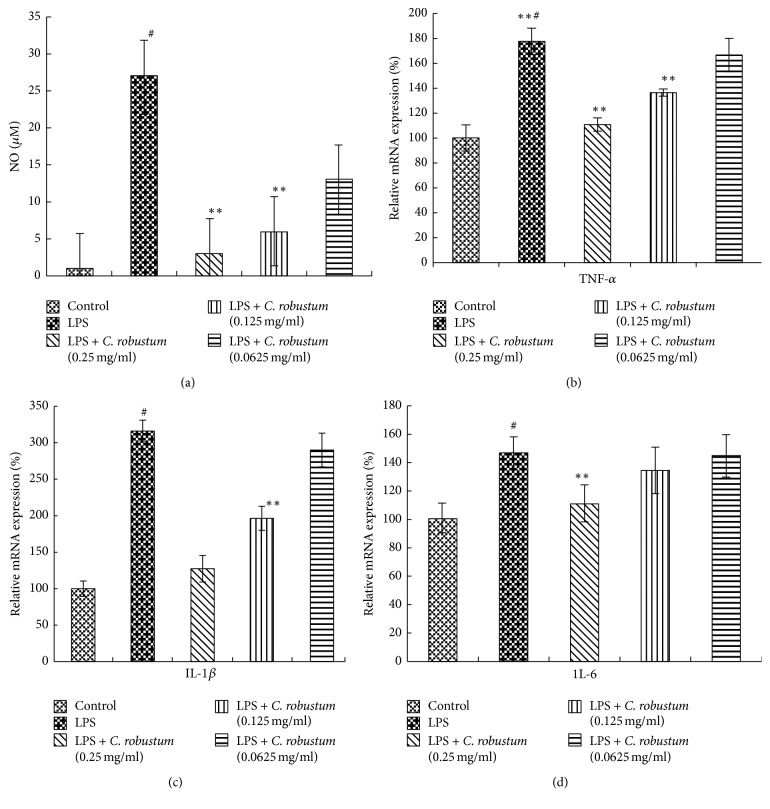
Effects of CRME on cytokines in RAW264.7 cells. The cells were pretreated with different concentrations (0.250, 0.125, or 0.063 mg/mL) of CRME for 1 h and then continuously incubated with 1 *μ*g/mL LPS for 24 h. Inhibition of NO in RAW264.7 cell supernatant by Griess method. (a) Inhibition of NO in RAW264.7 cells supernatant by Griess method. (b) Real-time PCR showing the TNF-*α* mRNA expression in RAW264.7 cells. (c) Real-time PCR showing the IL-1*β* mRNA expression in RAW264.7 cells. (d) Real-time PCR showing the IL-6 mRNA expression in RAW264.7 cells. All values are expressed as the mean ± SEM from three independent experiments. Data were analyzed by two-way ANOVA, Student-Newman-Keuls test as post hoc test (^#^*P* < 0.01, in comparison with control; ^*∗∗*^*P* < 0.01, in comparison with LPS).

The CRME showed dose-dependent inhibition of the generation of NO in LPS-stimulated RAW264.7 cells (Figure 2(a)). This finding suggested that the CRME may suppress the LPS-induced inflammatory response through inhibition of NO generation. Therefore, this result showed that CRME has anti-inflammatory response.

### 3.2. Gene Expression of IL-1 *β*, IL-6, and TNF-*α* in Cell Culture

CRME exerted an anti-inflammatory effect on LPS-induced responses accompanied by the gene expression of proinflammatory cytokines. RAW264.7 cells were treated with CRME and LPS for 24 h. The gene expression levels of TNF-*α*, IL-1*β*, and IL-6 were reduced by treatment with CRME in a dose-dependent manner (Figures 2(b), 2(c), and 2(d)). These results indicated that CRME has an anti-inflammatory effect on the gene expression of LPS-induced proinflammatory cytokines in RAW264.7 cells.

### 3.3. Morphologic Study of Survival State of Mice

**Figure 3:**
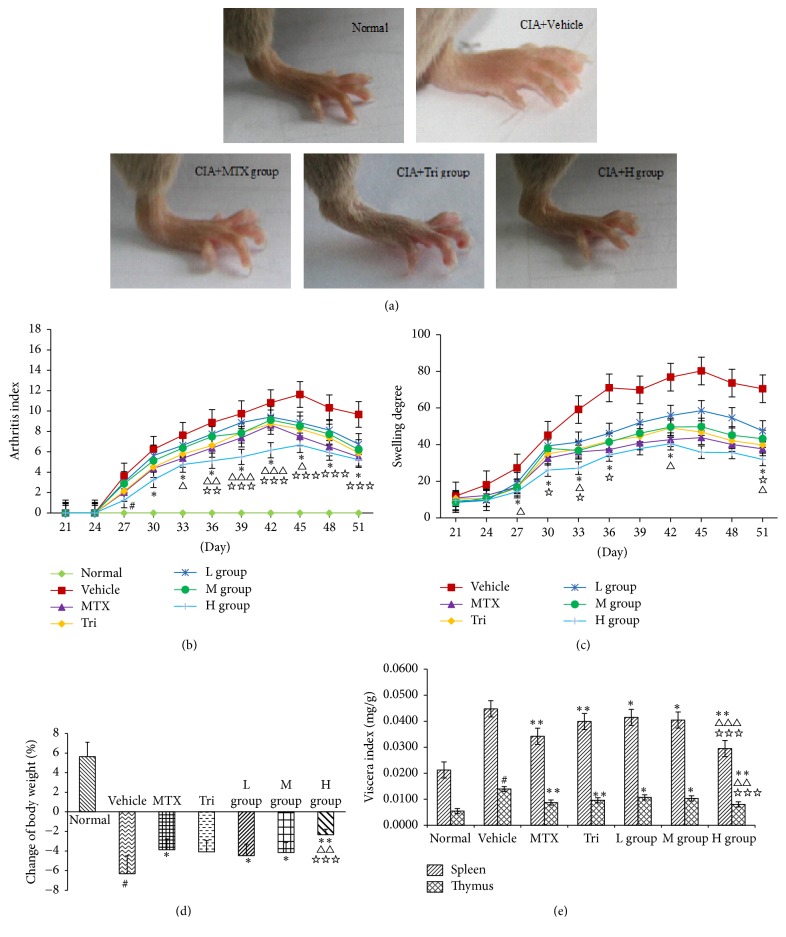
(a) Effect on CRME on disease progression in hind paw swelling in mice with CIA-induced arthritis. (A) Normal group, (B) CIA + vehicle, (C) CIA + MTX, (D) CIA + Tri, and (E) CIA + H group (99.4 mg/kg). Effects of CRME, MTX, and Tri treatment on the severity of disease progression as evidenced by arthritic index (b) and swelling degree (c) are presented here. There was a significant suppression of both the arthritic index and swelling degree by CRME and positive drug treatment within days 27~51. CRME on severity of arthritis of CIA mice was evaluated through body weight (d) and visceral index (e). Data are expressed as the mean ± SD of *n* = 6 animals per group. Data were analyzed by two-way ANOVA and Student-Newman-Keuls test as post hoc test (^#^*P* < 0.01, compared with the normal group. ^*∗*^*P* < 0.05 and ^*∗∗*^*P* < 0.01, compared with vehicle group. ^△^*P* < 0.05, ^△△^*P* < 0.01, and ^△△△^*P* < 0.001, compared with MTX group. ^☆^*P* < 0.05, ^☆☆^*P* < 0.01, and ^☆☆☆^*P* < 0.001, compared with Tri group).

Approximately 7 days after the secondary immunization, compared with the normal group, joints of mice in the vehicle group appeared obviously swollen and red, which indicated the success of the modeling. Starting from the 10th day, arthritis indexes were significantly lower for the CIA mice in each administered group than for the model group (Figure 3(a)).

As shown in Figure 3(b), the arthritis index of the H group had a significant difference from vehicle group (*P* < 0.01) starting from the 13th day, and with the passage of time, differences between the H group and positive medicine group increased gradually with statistical significance (All *P* < 0.05). As shown in Figure 3(c), the limb swelling degree of CIA mice in the H group was significantly lower than that in the positive medicine group (All *P* < 0.05). On day 51, the CIA swelling degree of the H group had dropped to 29.8%.

Remarkably, CRME could effectively suppress the loss of body weight of CIA mice (for the L, M, and H groups versus vehicle: *P* < 0.05). The weight loss of the H group was obviously lower than that of MTX and Tri, and the differences were statistically significant (All *P* < 0.05) (Figure 3(d)). In addition, the spleen index and thymus index of mice were lower in the CRME groups and positive drug groups than in the vehicle group, with significant differences (All *P* < 0.01) (Figure 3(e)).

### 3.4. Analysis of Histopathology

**Figure 4:**
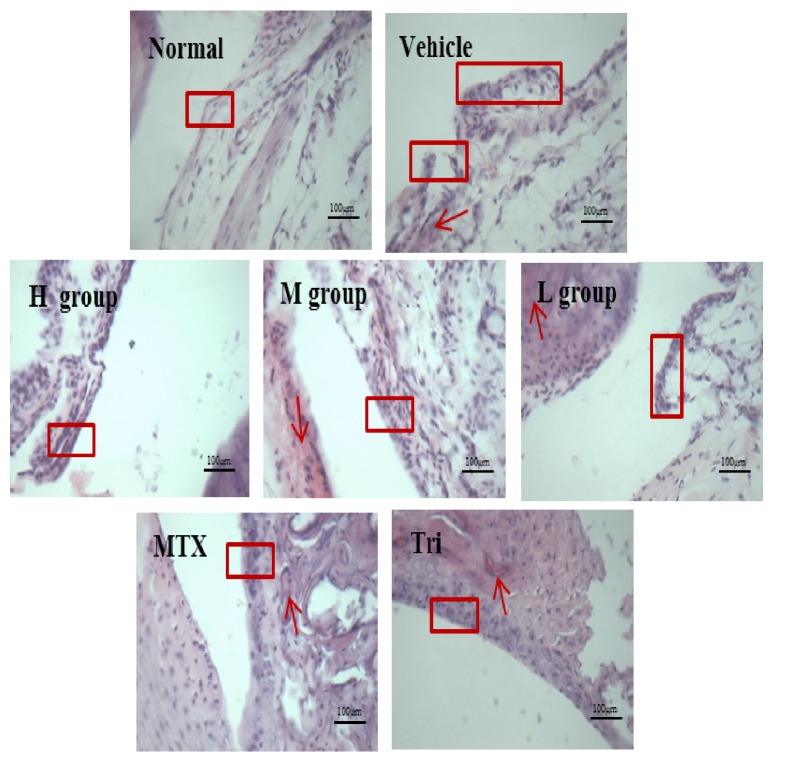
Influence of CRME on CIA-induced histopathological changes in the left hind limbs and immune organs of CIA-induced arthritic mice. A number of expanded vascular arteries and veins (arrows ↓) are indicated in the red boxes. HE stain, scale bars = 400 nd.

Joint pathological results (Figure 4) showed that synovial membrane hyperplasia in the normal group had not been found, its cell nuclear and cytoplasmic staining were normal, and loose underlying connective tissue was seen clearly; vascular arteries and veins showed no expansion and no blood stasis or inflammatory cell infiltration. Compared with the normal group, the synovial membrane of the vehicle group was thick, partly falling off, and uneven. It had a large number of inflammatory cells; at the same time, it was accompanied by capillary expansion. While compared with vehicle group, the synovium of CIA mice had thinned, with neat edges. The number of inflammatory cells was reduced, and capillary expansion was not obvious. Expansion of vascular arteries and veins and blood stasis were less than in the MTX (0.9 mg/kg) and Tri groups (11.3 mg/kg).

Results of histology of the left hind limbs and immune organs. At the end of the experiment, the hind limbs and immune organs of CIA mice were examined for histology. Mice in H group were orally given 99.4 mg/kg CRME. Pathological sections of joints (a part of the synovial cells in the red box, with arrows showing vascular artery and vein expansion) are shown in Figure 4.

### 3.5. Analysis of Proinflammatory Cytokines

**Figure 5:**
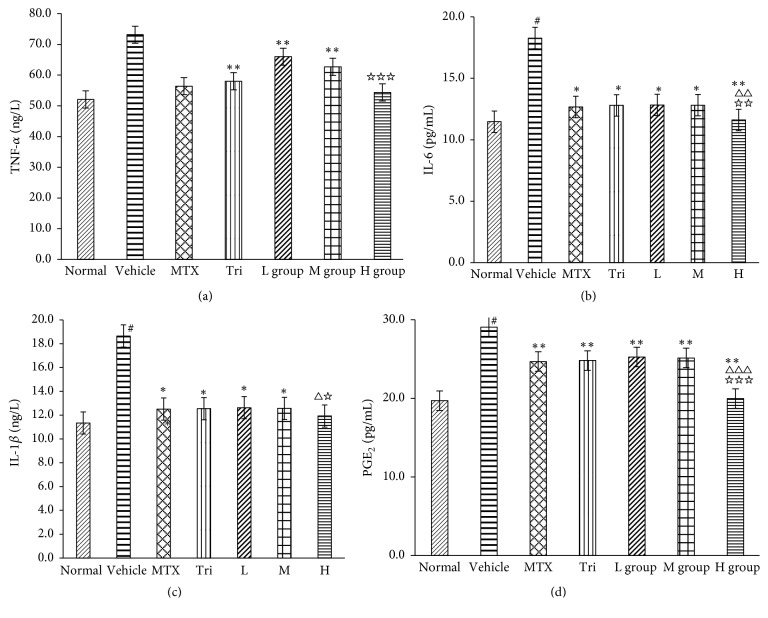
Effect of CRME on the secretion of proinflammatory mediators in CIA mice. The levels of TNF-*α* (a), IL-6 (b), IL-1*β* (c), and PGE2 (d) were monitored in the medium by using ELISA. Data represent the mean ± SD of three separate experiments. Data were analyzed by two-way ANOVA and Student-Newman-Keuls test as post hoc test (^#^*P* < 0.01, compared with the normal group. ^*∗*^*P* < 0.05 and ^*∗∗*^*P* < 0.01, compared with vehicle group. ^△^*P* < 0.05, ^△△^*P* < 0.01, and ^△△△^*P* < 0.001, compared with MTX group. ^☆^*P* < 0.05, ^☆☆^*P* < 0.01, and ^☆☆☆^*P* < 0.001, compared with Tri group).

TNF-*α*, IL-1*β*, IL-6, and PGE2 levels are indicated in Figure 5. As shown in the results, the levels of IL-1*β*, IL-6, TNF-*α*, and PGE2 in sera of CIA mice were apparently higher in the vehicle group than the normal group, differences that were statistically significant (All *P* < 0.01). A significant decrease in TNF-*α*, IL-1*β*, IL-6, and PGE2 levels was observed in all administered groups (H, L, and M group) when compared to the vehicle group. In addition, the levels of IL-1*β*, IL-6, TNF-*α*, and PGE2 of CIA mice were distinctly lower in the H group than in the MTX group or Tri group (*P* < 0.05), while the differences among the L group, M group, and positive drug groups were not statistically significant (*P* > 0.05) (Figure 5).

### 3.6. Analysis of Immunohistochemistry

**Figure 6:**
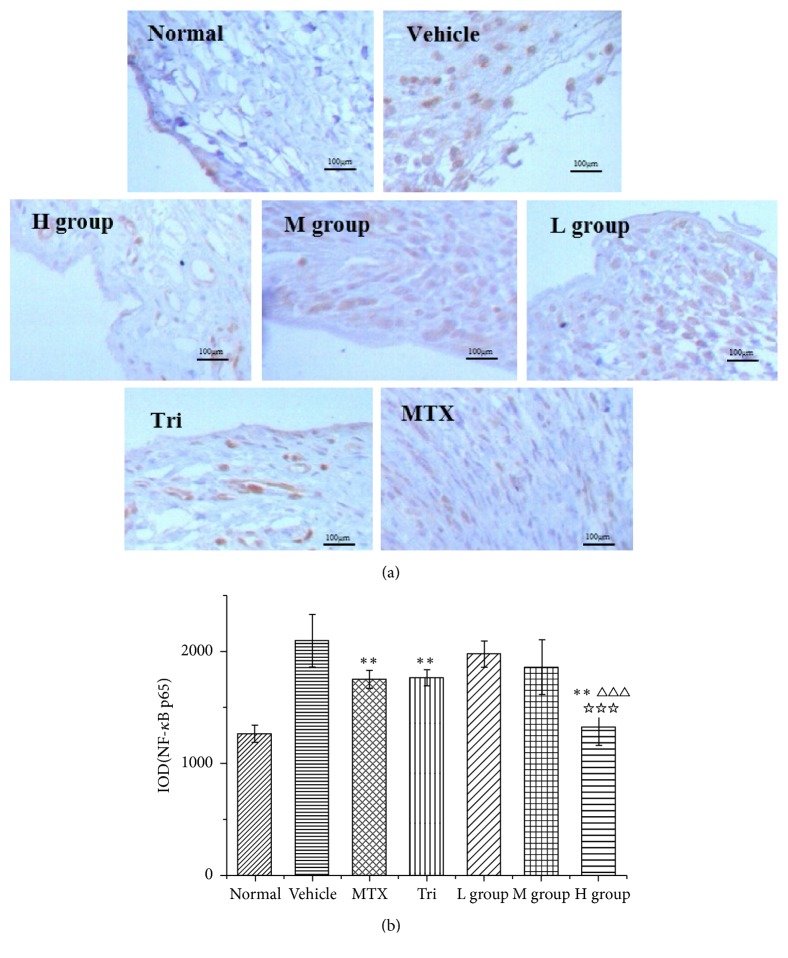
Analysis of immunohistochemistry of the right hind limbs. After the intervention, mice were sacrificed, and, then, the joints were subjected to immunohistochemistry for NF-*κ*B p65. (a) NF-*κ*B p65; (b) IOD of NF-*κ*B p65. The data are presented as the mean ± SD with *n* = 6 mice per group. Data were analyzed by two-way ANOVA, Student-Newman-Keuls test as post hoc test (^*∗*^*P* < 0.05 and ^*∗∗*^*P* < 0.01, compared with the vehicle group. ^△△△^*P* < 0.001, compared with the MTX group. ^☆^*P* < 0.05 and ^☆☆☆^*P* < 0.001, compared with the Tri group). HE stain, scale bars = 400 nd.

As shown in Figure 6(a), the NF-*κ*B p65 positive expression parts are tan or brown particles. Data analysis indicated in Figure 6(b) shows that NF-*κ*B p65 is expressed more positively in vehicle group than in treatment group (*P* < 0.05). What is more, the IOD volume of NF-*κ*B p65 in CIA mice from the H group was 1325.18, which was lower than that from the positive drug group (Tri: 1765.43, MTX: 1751.42) (*P* < 0.001).

### 3.7. Discussion

Thus, it may be functionally important to tightly regulate the degree of phlogistic pathway activation, which could be a good therapeutic target to resist RA. For the last decade, efforts have been made to develop these agents. However, the long-term administration of anti-inflammatory drugs is limited due to the side effects. Now, there are biological inhibitors such as TNF-*α* inhibitors and infliximab [19, 20]. These activators can also induce specific immunity to increase the self-protection ability of the organism. An excessive inflammatory reaction can also damage target cells and tissue. Therefore, novel anti-RA agents with fewer side effects are needed. TCM can offer a promising repertoire of potentially therapeutic agents for RA [21].* C. robustum* has often been used for treating RA patients in China. In books, such as the compendium of Chinese traditional herbal medicine, it has been recorded that* C. robustum* tincture can be made with 15 g of roots and rhizomes soaked in 300 mL of alcohol for 7 days, and this was used to treat joint pain, bruises, and RA. In this paper, we found that CRME possessed inhibitory activity against NO, IL-1*β*, IL-6, and TNF-*α* in RAW267.4 cells stimulated by LPS.

Our present data show that all the doses of CRME (24.8, 49.6, and 99.4 mg/kg) significantly inhibited edema compared to their untreated counterparts and with greater effect at 99.4 mg/kg than Tri at 11.3 mg/kg. In reducing the loss of body weight, CRME also has an advantage over MTX and Tri. Then, CRME improved histological findings by decreasing the extent of inflammatory cell infiltration and the capillary expansion of CIA mice more effectively than MTX and Tri. These results were also confirmed by the expression of inflammatory cytokines by ELISA, which showed that TNF-*α*, IL-6, IL-1*β*, and PGE2 were distinctly lower than in the MTX group and Tri group. The results showed that oral administration of CRME may effectively preserve both synovial membrane damage and inflammatory cell infiltration of inflamed joints. This clearly showed the recuperative RA function and lower toxicity of CRME at the given dosages. However, it should be noted that long-term intake of high concentration of CRME (1.59 g/kg) will show gastrointestinal irritation and vomiting from our previous study. Furthermore, Kennelly et al. showed that the alkaloid contained in the* C. robustum* had a potential teratogenic effect in mice embryonic culture experiments in vitro [12]. It has also been reported that Cauloside B and Cauloside C had cytotoxicity on the growth of sea urchin embryos by altering cell permeability [13, 14]. Kawai et al. studies have shown that Caulosides A~C have a high hemolytic effect [22]. Therefore, patients with gastrointestinal diseases and pregnant women should not use CRME.

*C. robustum* consists of more than 60 constituents, including sterols, triterpenes, glycosides, and alkaloids. Triterpene saponins are believed to be the major bioactive compounds contributing to the anti-inflammatory effects on RA [10]. Triterpene saponins constitute an important class of potential pharmacological agents possessing a range of different physiological activities including anticancer, anticoagulation, anti-inflammation, antibacterial, analgesic, and comparative immune-modulation activity [23]. Recently, triterpene saponins have attracted positive research interest. Of great interest is the possibility that this class of constituents could be a source of drugs for the treatment of several diseases. We investigated the triterpene saponin content in all CRME. Our findings suggest that CRME is mainly composed of six marker compounds including Cauloside H, Leonticin D, Cauloside G, Cauloside D, Cauloside B, and Cauloside C, which constitute more than 50 percent. In particular, the abovementioned compounds seem to act as inhibitors of inflammatory factors [24].

The inhibition of NF-*κ*B might result in a marked amelioration of chronic inflammation by suppressing the production of cytokines [25]. Measurable levels of cytokines such as TNF-*α*, IL-6, IL-1*β*, and PGE2 are found locally in synovial tissue, the major site of inflammation in CIA as well as RA, where they facilitate the local and systemic inflammatory response [26, 27]. Existing research showed that the NF-*κ*B in synovial cells induced by TNF-*α* can mediate nuclear transfer leading to NF-*κ*B signaling pathway activation [28]. To be more specific, to activate the NF-*κ*B pathway, TNF-*α* has a variety of signal molecules involved, including TNFR related factor, RIP (receptor interacting protein), MAP3K (mitogen activated protein kinase 3), and IKK compounds [29]. In turn, TNF-*α* produces physiological reaction via NF-*κ*B acting as an important mediator, and activated NF-*κ*B can also induce the expression of TNF-*α* and enhance TNF-*α* gene transcription. In some cells and tissues, NF-*κ*B p65 and TNF-*α* both promote each other, which creates a vicious cycle that increases RA. In RA, IL-6 mainly induces liver cell synthesis of a variety of acute phase reaction proteins and promotes the synthesis of B cells to produce immunoglobulin and rheumatoid factor [30]. It has been confirmed that NF-*κ*B can promote gene transcription of inflammation factors such as TNF-*α*, IL-6, and IL-1*β* [31]. In addition, it might enhance the inflammatory damage of TNF-*α* and IL-1 by improving the generation and release of TNF-*α* and IL-1*β* [32]. IL-1*β* is of importance in passing information and mediating immune cell activation, proliferation, and inflammatory response. IL-1*β*, acting as another important extracellular cue, can activate NF-*κ*B to amplify the inflammatory response. NF-*κ*B is promoted to transport into the nucleus by IL-1*β*, where it combines with the promoters of target genes to start target protein expression [33]. As one of the most important targets for the treatment of inflammation and tumor molecules, PGE2 can promote the division and proliferation of fibroblasts and induce microvascular formation and growth [34]. Like TNF-*α*, IL-6, and IL-1*β*, PGE2 participates in synovium and joint inflammation of surrounding tissue, cartilage destruction, and hyperplasia lesions through the NF-*κ*B signaling pathway [35].

We confirmed that the pathogenesis of CIA was accompanied by a substantial increase in the levels of TNF-*α*, IL-6, IL-1*β*, and PGE2 in the plasma of mice. Interestingly, the administration of CRME drastically reduced the levels of these four proinflammatory cytokines significantly. Our results indicated that CRME effectively improved arthritic symptoms including limb swelling by downregulating the production of proinflammatory cytokines. Further, we suspect that CRME, being an important negative regulator of these proinflammatory cytokines related to RA, might work by suppressing the expression of NF-*κ*B.

## 4. Conclusions

It has been thoroughly demonstrated that CRME is an anti-inflammatory drug as observed under morphological study in CIA mice. This was evident from not only the significantly improved symptoms of the arthritis index and limb swelling but also the improved histological findings of decreased synovial membrane damage and inflammatory cell infiltration in CIA mice. We also revealed that the protective mechanisms of CRME treatment could be partially explained by a decrease in the proinflammatory cytokines TNF-*α*, IL-6, IL-1*β*, and PGE2. Specifically, it has been found to be very effective at ameliorating the damage resulting from progression of arthritis by controlling the expression of NF-*κ*B. Additional studies are required with more physiological and pharmaceutical investigations to establish its clinical applicability.
